# Clinical utility of the AITIS model for test-free identification of sarcopenia in patients with stage IV–V non-dialysis-dependent chronic kidney disease

**DOI:** 10.3389/fnut.2026.1793383

**Published:** 2026-04-28

**Authors:** Yang Li, Youying Zhang, Daxiang Hu, Xiaoyuan Li, Mengda Tang, Yu Cao, Jiachuan Xiong, Jinghong Zhao, Liangyu Yin

**Affiliations:** 1 Department of Nephrology, Chongqing Key Laboratory of Prevention and Treatment of Kidney Disease, Chongqing Clinical Research Center of Kidney and Urology Diseases, Xinqiao Hospital, Army Medical University (Third Military Medical University), Chongqing, China; 2 Lead Contact and Principal Investigator of the Multidimensional Nutritional Status and Clinical Outcomes in Chronic Kidney Disease (MNSCOC) Project, Chongqing, China

**Keywords:** artificial intelligence, chronic kidney disease, diagnosis, machine learning, prediction, sarcopenia

## Abstract

**Background:**

Identifying sarcopenia in resource-limited settings presents a significant challenge. The objective of this study was to validate the clinical usefulness of the Artificial Intelligence to Identify Sarcopenia (AITIS) model, a test-free artificial intelligence model we previously proposed for identifying sarcopenia, in patients with chronic kidney disease (CKD).

**Methods:**

This observational cross-sectional study enrolled 236 patients with stage IV–V CKD. Sarcopenia was diagnosed using the Asian Working Group for Sarcopenia 2019 criteria, which are based on handgrip strength, physical performance, and appendicular skeletal muscle mass measured by bioelectrical impedance analysis. Patient data, including age, sex, height, weight, and 20 functional measures, were used as predictors. The AITIS model was applied to predict sarcopenia, and its performance, explainability, and clinical usefulness were comprehensively analyzed.

**Results:**

The study included 129 men and 107 women (median age = 54.5 years). Sarcopenia was diagnosed in 62 patients (26.3%). The three most common functional limitations reported were jogging 1 km (*n* = 77, 32.6%), climbing stairs (*n* = 56, 23.7%), and walking 1 km (*n* = 13, 5.5%). In contrast, no difficulty was reported in getting in and out of bed, using the toilet, or controlling urination and defecation. The AITIS model demonstrated favorable performance in predicting sarcopenia within the study population [AUC (95% CI) = 0.792 (0.729–0.856), area under the precision-recall curve = 0.610, kappa = 0.398, accuracy (95% CI) = 0.758 (0.699–0.812), sensitivity = 0.597, specificity = 0.816]. Calibration curve analysis revealed good agreement between model predictions and the ground truth. Model performance improved in patients with less physical activity (AUC = 0.843, 95% CI = 0.764–0.922) and in those with stage IV CKD (AUC = 0.824, 95% CI = 0.739–0.909). The SHAP summary plot indicated that age, walking 1 km, and lifting 5 kg were the top three contributors to the model’s predictive ability. Decision curve analysis supported the model’s clinical usefulness.

**Conclusion:**

The AITIS model demonstrates strong generalizability and performance in predicting sarcopenia in patients with stage IV–V CKD. These findings may enhance clinical decision-making and facilitate the development of novel strategies for managing sarcopenia in CKD patients.

## Introduction

Chronic kidney disease (CKD) is a highly prevalent condition defined by persistent alterations in kidney structure and/or function, which have multiple health implications (1). CKD affects over 10% of the global population (2) and 8.2–10.8% of the Chinese population (3, 4), significantly increasing the burden of cardiovascular diseases, other morbidities, and mortality (3). Despite optimal treatment, CKD can still progress to end-stage renal disease (5). Therefore, current clinical guidelines emphasize multidisciplinary management strategies to minimize the health complications and adverse outcomes associated with CKD (6).

Patients with CKD often face elevated risks such as protein-energy wasting, chronic inflammation, metabolic acidosis, insulin resistance, and hormonal disturbances (1, 6, 7). These factors can contribute to the loss of muscle mass and/or function in CKD patients, collectively known as sarcopenia (8–10). The global prevalence of sarcopenia across the broad spectrum of CKD is approximately 24.5% (11), and can be as high as 75.6% in peritoneal dialysis patients (12). Sarcopenia not only leads to decreased physical function and reduced quality of life but is also closely associated with an increased risk of falls, fractures, higher hospitalization rates, elevated cardiovascular events, and all-cause mortality (13–15). Notably, since sarcopenia and its components can potentially be reversed through noninvasive interventions such as physical exercise (16), early identification of sarcopenia in patients with CKD has become imperative to facilitate timely interventions (17).

Two widely used diagnostic frameworks for sarcopenia include the European Working Group on Sarcopenia in Older People (EWGSOP2) for Western populations (10) and the 2019 consensus update of the Asian Working Group for Sarcopenia (AWGS) for Asian populations (9). Both frameworks adopt a similar diagnostic workflow, which includes the assessment of muscle strength, skeletal muscle mass, and physical performance. Although these parameters are reliable, they must be evaluated under the supervision of medical professionals, necessitating that patients visit healthcare institutions in person. More importantly, measuring skeletal muscle mass requires equipment such as dual-energy X-ray absorptiometry (DEXA) or bioelectrical impedance analysis (BIA), which poses challenges for smaller institutions and community or home settings that may lack the necessary resources. Consequently, some patients may remain undetected, missing the optimal window for intervention.

To address these challenges, we previously proposed an artificial intelligence model named the Artificial Intelligence to Identify Sarcopenia (AITIS). Its distinctive feature is that it requires no measurements of muscle strength, mass, or physical performance, nor does it require the involvement of healthcare professionals. Participants only need to provide information such as age, sex, height, weight, and answers to questions related to their daily activity abilities to complete a rapid screening for sarcopenia risk. In preliminary development and validation studies, the model demonstrated excellent performance in predicting sarcopenia risk. However, AITIS was developed based on a community-dwelling population of middle-aged and older adults (18), among whom only a proportion had CKD (19–21). Therefore, whether this model can be applied in the context of CKD remains unclear. This study aims to systematically validate the feasibility of AITIS for identifying sarcopenia in patients with stage IV–V CKD to facilitate the development of novel management strategies for sarcopenia in this population.

## Methods

### Study design and population

This was a single-center, observational cross-sectional study. Patients were drawn from an ongoing project, the Multidimensional Nutritional Status and Clinical Outcomes in Chronic Kidney Disease (MNSCOC), which is registered online (https://www.chictr.org.cn/showproj.html?proj=298129, ID: ChiCTR2600116073). The MNSCOC project is designed as an ambispective cohort study, focusing on nutrition-related health issues and clinical outcomes in patients with CKD. Patients in the present study were derived from the retrospective part of the MNSCOC database. The inclusion criteria for the present study were as follows: (1) patients diagnosed with stage IV–V CKD according to the KDIGO international guidelines (6); (2) age ≥18 years; (3) patients who were not receiving renal replacement therapy; and (4) patients with available data on age, sex, height, weight, sarcopenia-related data, activities of daily living (ADL), instrumental activities of daily living (IADL), and other functional capacity (FC) data. The exclusion criteria included: (1) presence of a cardiac pacemaker; (2) comorbid malignancies; (3) conditions such as thyroid dysfunction, liver diseases, or acute infections; (4) severe cognitive impairment; and (5) patients assessed by clinicians as having potentially inaccurate body composition analysis results (e.g., those with significant edema or ascites). Initially, we included 249 patients from August 2024 to March 2025. Exclusions were made for patients who missed the required body component analysis (BCA, *n* = 2) and FC assessments (*n* = 1), those who had duplicate enrollments (*n* = 5), and those with outlier values (*n* = 5). This left 236 patients for formal analysis (a flowchart of patient inclusion is shown in Supplementary Figure S1). The MNSCOC project was approved by the Medical Ethics Committee of the Second Affiliated Hospital of Army Medical University (Approval No. 2025-Yan-433-01). All data were de-identified prior to analysis, and the principles of the Declaration of Helsinki were followed.

### Data acquisition and handling

Data were obtained through in-person interviews and physical examinations conducted by a project-trained researcher within the first 24 h after each patient’s admission to our hospital. The collected data included patient age (years), sex, body height (cm), body weight (kg), smoking status (never, current, or former), and alcohol consumption (defined as drinking at least once a week in the past year). Other recorded variables included physical exercise (defined as regular exercise at least once a week in the past 3 months), residency (urban vs. rural), education level (≥ high school vs. lower), occupation (white-collar vs. others), marital status (married vs. others), and comorbidities (hypertension, diabetes, hyperlipidemia, and anemia). Body mass index (BMI, kg/m^2^) was calculated as weight in kilograms divided by height in meters squared and was categorized as underweight (<18.5), normal (18.5 to <24), overweight (24 to <28), or obese (≥28) according to Chinese recommendations (22). All serum indices were measured in the clinical laboratory of our hospital using fasting blood samples drawn upon admission. These indices included creatinine, albumin, triglycerides, total cholesterol, high-density lipoprotein cholesterol, low-density lipoprotein cholesterol, glucose, and hemoglobin. ADL, IADL and other FC variables were analyzed using a 20-item questionnaire in accordance with the AITIS study (18). The 20 FC items comprised 6 ADLs (dressing, bathing, eating, bed mobility, toileting, and urination), 5 IADLs (managing money, medication management, shopping, meal preparation, and housework), and 9 other FC items (jogging 1 km, walking 1 km, walking 100 m, sitting in a chair, climbing stairs, stooping, lifting 5 kg, picking up objects, and reaching). Detailed descriptions of the 20 FC items are provided in Supplementary Table S1. Difficulty with any task was scored as 1 point (positive), while no difficulty was scored as 0 points (negative).

### Diagnosis of sarcopenia

Sarcopenia was prospectively diagnosed based on the AWGS 2019 framework (9). According to the AWGS, sarcopenia is diagnosed when there is low appendicular skeletal muscle mass index (ASMI) combined with low handgrip strength (HGS) and/or reduced physical performance. Positivity for all three criteria indicates severe sarcopenia. HGS was measured using an electronic hand grip dynamometer (Model: EH101, Guangdong Senssun Weighing Apparatus Group Ltd., Guangdong, China). Participants were instructed to stand with their arms relaxed and perform three maximal squeezes; the highest value was recorded. Low HGS was defined as <28 kg for men and <18 kg for women (9). A trained researcher conducted body composition analysis using a device (Model S10, Inbody, Seoul, Korea) according to the manufacturer’s instructions to measure the appendicular skeletal muscle mass (ASM) for each patient. ASMI was then calculated as ASM in kilograms divided by height in meters squared (kg/m^2^). Physical performance was assessed using the chair stand test (CST). Patients were instructed to perform five consecutive stand-sit movements at their fastest pace, with their arms folded across their chest to prevent using them for assistance. The total time required to complete the five movements was recorded in seconds, with a completion time of ≥12 s indicating low physical performance (9).

### Prediction of sarcopenia using the AITIS model

The AITIS model was deployed as a web application^1^ (18). Patients’ data, including age, sex, height, weight, and 20 FC items, were input into the user interface of the web application. The predicted class (sarcopenia vs. not sarcopenia) and the probability of each class (ranging from 0 to 1) displayed on the website were recorded. We also performed local batch predictions using the model file,^2^ which returned results consistent with those from the web application.

### Statistical analysis

Continuous variables are presented as medians (interquartile ranges) and compared using the Wilcoxon rank-sum test. Categorical variables are expressed as numbers (percentages) and compared using the chi-squared test. Model performance evaluation metrics included area under the curve (AUC), kappa coefficient, accuracy (95% CI), sensitivity, specificity, positive predictive value (PPV), and negative predictive value (NPV), as these are commonly used in clinical applications. The consistency between model predictions and the ground truth was evaluated using a calibration curve. The SHapley Additive exPlanations (SHAP) method was employed to assess feature importance and model explainability within the study population. Decision curve analysis was conducted to evaluate the clinical usefulness of the model. All reported *p*-values were two-sided and considered significant at *p* < 0.05. All statistical analyses were performed using R (version 4.3.1, Foundation for Statistical Computing, Vienna, Austria) or Python (version 3.9.11, The Python Software Foundation, United States).

## Results

### Population overview

The characteristics of the study population are shown in the overall column of Table 1. There were 236 patients with a median age of 54.5 years, including 129 men and 107 women. Among them, 10 (4.2%), 115 (48.7%), 76 (32.2%), and 35 (14.8%) patients were classified into the underweight, normal weight, overweight, and obese groups, respectively. The prevalence of low HGS, low ASMI, and poor physical performance (indicated by the CST) was 105 (44.5%), 23 (9.7%), and 69 (29.2%), respectively. The most frequently reported functional limitations were observed for jogging 1 km (*n* = 77, 32.6%), climbing stairs (*n* = 56, 23.7%), walking 1 km (*n* = 13, 5.5%), and doing housework (*n* = 10, 4.2%). In contrast, no difficulty was reported for getting in and out of bed, using the toilet, or controlling urination and defecation.

**Table 1:** Baseline characteristics of the study population by sarcopenia.

		Sarcopenia		
Characteristics	Overall (*n* = 236)	No (*n* = 174)	Yes (*n* = 62)	*p*
Demographics				
Age, years	54.5 [45.0, 63.0]^a^	51.5 [43.0, 59.0]	63.0 [56.0, 70.8]	<0.001
Sex, men	129 (54.7)^b^	97 (55.7)	32 (51.6)	0.680
Height, cm	162.0 [156.8, 168.0]	163.0 [158.0, 168.0]	160.0 [155.0, 163.8]	0.001
Weight, kg	62.0 [55.0, 70.2]	63.0 [57.5, 72.0]	56.9 [52.0, 67.6]	0.002
BMI, kg/m^2^	23.6 [21.4, 26.5]	23.9 [21.7, 26.6]	22.8 [20.6, 25.8]	0.073
BMI category				0.389
Underweight	10 (4.2)	8 (4.6)	2 (3.2)	
Normal	115 (48.7)	79 (45.4)	36 (58.1)	
Overweight	76 (32.2)	59 (33.9)	17 (27.4)	
Obese	35 (14.8)	28 (16.1)	7 (11.3)	
Smoking				0.677
None smoker	141 (59.7)	104 (59.8)	37 (59.7)	
Current smoker	49 (20.8)	38 (21.8)	11 (17.7)	
Former smoker	46 (19.5)	32 (18.4)	14 (22.6)	
Drinking, yes	31 (13.1)	26 (14.9)	5 (8.1)	0.247
Physical exercise, mod-rig, m/day	20.0 [0.0, 40.0]	20.0 [0.0, 40.0]	0.0 [0.0, 37.5]	0.400
Residency, urban	188 (79.7)	135 (77.6)	53 (85.5)	0.253
Education level, ≥high school	86 (36.4)	70 (40.2)	16 (25.8)	0.061
Occupation, white-collar	38 (16.1)	33 (19.0)	5 (8.1)	0.071
Marriage, married	208 (88.1)	156 (89.7)	52 (83.9)	0.327
Comorbidities				
Hypertension, yes	150 (63.6)	110 (63.2)	40 (64.5)	0.977
Diabetes, yes	60 (25.4)	39 (22.4)	21 (33.9)	0.108
Hyperlipidemia, yes	84 (35.6)	63 (36.2)	21 (33.9)	0.861
Anemia, yes	113 (47.9)	81 (46.6)	32 (51.6)	0.591
Clinical parameters				
CKD stage, V vs. IV	114 (48.3)	83 (47.7)	31 (50.0)	0.870
Creatinine, μmol/L	329.5 [233.2, 500.8]	336.0 [227.2, 506.2]	319.5 [245.8, 490.8]	0.865
Albumin, g/L	40.4 [35.5, 43.3]	41.0 [35.8, 43.5]	39.2 [34.6, 42.6]	0.083
Triglycerides, mmol/L	1.6 [1.1, 2.3]	1.5 [1.1, 2.2]	1.6 [1.1, 2.3]	0.759
Total cholesterol, mmol/L	4.3 [3.6, 5.1]	4.2 [3.6, 5.0]	4.3 [3.5, 5.4]	0.372
HDL-c, mmol/L	1.2 [1.0, 1.5]	1.2 [1.0, 1.4]	1.3 [1.0, 1.6]	0.374
LDL-c, mmol/L	2.5 [2.0, 3.2]	2.5 [2.0, 3.2]	2.5 [2.0, 3.2]	0.967
Fasting glucose, mmol/L	5.4 [4.9, 5.8]	5.3 [4.9, 5.8]	5.5 [5.1, 6.1]	0.155
Hemoglobin, g/L	105.0 [93.0, 115.2]	107.0 [96.0, 117.0]	99.5 [83.0, 111.5]	0.002
Sarcopenia components				
Handgrip strength, kg	23.4 [19.2, 29.9]	25.3 [21.8, 33.8]	16.7 [15.4, 22.8]	<0.001
Handgrip strength, low	105 (44.5)	43 (24.7)	62 (100.0)	<0.001
ASMI, kg/m^2^	7.4 [6.5, 8.2]	7.4 [6.6, 8.4]	6.7 [6.0, 7.7]	<0.001
ASMI, low	23 (9.7)	7 (4.0)	16 (25.8)	<0.001
Five-time chair stand test, s	10.4 [8.9, 12.3]	9.8 [8.5, 11.1]	14.1 [12.3, 16.6]	<0.001
Five-time chair stand test, low	69 (29.2)	20 (11.5)	49 (79.0)	<0.001
Functional capacity				
Dressing	1 (0.4)	0 (0.0)	1 (1.6)	0.589
Bathing	2 (0.8)	0 (0.0)	2 (3.2)	0.116
Eating	1 (0.4)	0 (0.0)	1 (1.6)	0.589
Bed	0 (0)	0 (0)	0 (0)	NA
Toilet	0 (0)	0 (0)	0 (0)	NA
Urination	0 (0)	0 (0)	0 (0)	NA
Money	2 (0.8)	1 (0.6)	1 (1.6)	1.000
Medication	2 (0.8)	1 (0.6)	1 (1.6)	1.000
Shopping	3 (1.3)	1 (0.6)	2 (3.2)	0.347
Meal	5 (2.1)	2 (1.1)	3 (4.8)	0.223
Housework	10 (4.2)	4 (2.3)	6 (9.7)	0.035
Jogging 1 km	77 (32.6)	40 (23.0)	37 (59.7)	<0.001
Walking 1 km	13 (5.5)	4 (2.3)	9 (14.5)	0.001
Walking 100 m	3 (1.3)	0 (0.0)	3 (4.8)	0.024
Chair	2 (0.8)	0 (0.0)	2 (3.2)	0.116
Climbing	56 (23.7)	28 (16.1)	28 (45.2)	<0.001
Stooping	6 (2.5)	2 (1.1)	4 (6.5)	0.071
Lifting 5 kg	9 (3.8)	2 (1.1)	7 (11.3)	0.001
Picking	1 (0.4)	0 (0.0)	1 (1.6)	0.589
Arm	2 (0.8)	1 (0.6)	1 (1.6)	1.000

BMI, body mass index; mod-rig, moderate to rigor; CKD, chronic kidney disease; HDL-c, high density lipoprotein cholesterol; LDL-c, low density lipoprotein cholesterol; ASMI, appendicular skeletal muscle mass index; NA, not applicable.

a Median [interquartile range], all such values.

b Number (percentage), all such values.

### Sarcopenia

Sarcopenia was diagnosed in 62 (26.3%) patients. Baseline characteristics of the study population, stratified by sarcopenia, are shown in Table 1. Sarcopenia was significantly associated with higher values or rates of age, CST time, and difficulties in completing daily activities (including doing housework, jogging 1 km, walking 1 km, walking 100 m, climbing stairs, and lifting 5 kg). Conversely, sarcopenia was associated with lower values or rates of body height, body weight, hemoglobin, HGS, and ASMI (all *p* < 0.05).

### Model performance evaluation

**Figure 1:**
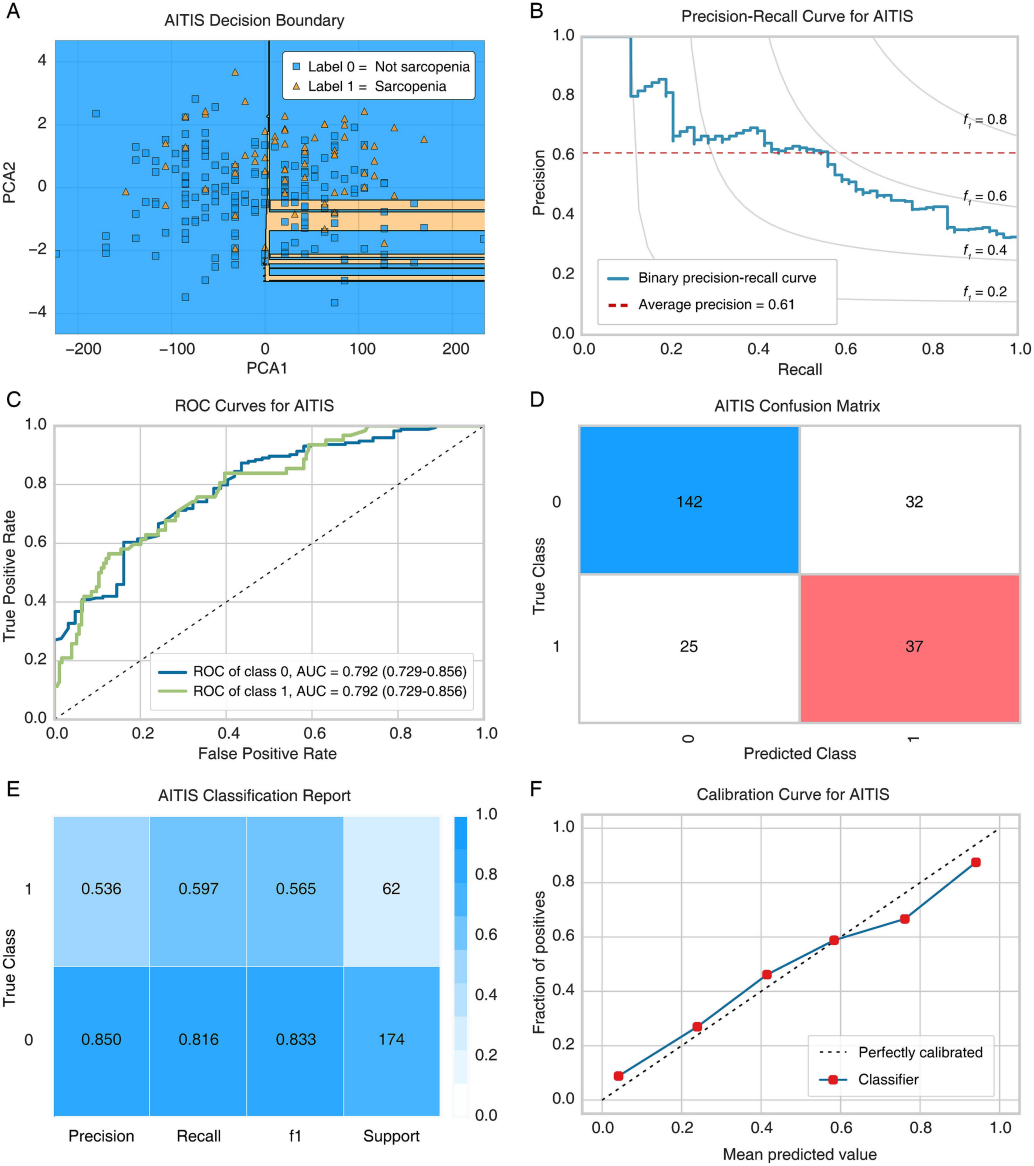
Evaluation of the AITIS model in the study population. **(A)** Decision boundary plot. **(B)** Precision-recall curve. **(C)** Receiver operating characteristic curve. **(D)** Confusion matrix. **(E)** Classification report. **(F)** Calibration curve. PCA, principle component analysis; ROC, receiver operating characteristic; AUC, area under the curve.

The evaluation of model performance is shown in Figure 1. The decision boundary of the AITIS model in the study population is demonstrated in Figure 1A. The precision-recall (PR) curve was shown in Figure 1B, with the area under the PR curve calculated at 0.610. The ROC curves showed that the AUC (95%CI) of the AITIS model was 0.792 (0.729–0.856) (Figure 1C). Analysis of the confusion matrix revealed that 142 patients (not sarcopenia) and 37 patients (sarcopenia) were correctly predicted (Figure 1D). The classification report showed that the precision, recall, and *F*_1_-score for the sarcopenia class were 0.536, 0.597, and 0.565, respectively. In contrast, these metrics were 0.850, 0.816, and 0.833 for the non-sarcopenia group (Figure 1E). The calibration curve of the AITIS model demonstrated good consistency between model predictions and actual observations (Figure 1F). Metrics for assessing the diagnostic method also support the feasibility of the AITIS model [kappa = 0.398, accuracy (95% CI) = 0.758 (0.699–0.812), sensitivity = 0.597, specificity = 0.816, PPV = 0.536, NPV = 0.850] (Table 2).

**Table 2:** Model performance in the overall study population and in different subgroups of interest.

Group	No./Events	AUC (95% CI)	Kappa	Accuracy (95% CI)	Sensitivity	Specificity	PPV	NPV
Overall	236/62	0.792 (0.729–0.856)	0.398	0.758 (0.699–0.812)	0.597	0.816	0.536	0.850
Age, years								
<60	156/21	0.690 (0.586–0.794)	0.162	0.821 (0.751–0.877)	0.238	0.911	0.294	0.885
≥60	80/41	0.713 (0.600–0.827)	0.270	0.637 (0.522–0.742)	0.780	0.487	0.615	0.679
Sex								
Men	129/32	0.796 (0.709–0.882)	0.393	0.783 (0.702–0.851)	0.500	0.876	0.571	0.842
Women	107/30	0.794 (0.701–0.888)	0.396	0.729 (0.634–0.810)	0.700	0.740	0.512	0.864
BMI								
Underweight/normal	125/38	0.789 (0.707–0.872)	0.395	0.744 (0.658–0.818)	0.579	0.816	0.579	0.816
Overweight/obese	111/24	0.804 (0.705–0.902)	0.399	0.775 (0.686–0.849)	0.625	0.816	0.484	0.888
Physical exercise, mod-rig								
<20 min/day	112/33	0.843 (0.764–0.922)	0.486	0.777 (0.688–0.850)	0.697	0.810	0.605	0.865
≥20 min/day	124/29	0.726 (0.625–0.827)	0.297	0.742 (0.656–0.816)	0.483	0.821	0.452	0.839
Occupation								
White-collar	38/5	0.733 (0.478–0.988)	0.371	0.868 (0.719–0.956)	0.400	0.939	0.500	0.912
Others	198/57	0.784 (0.717–0.852)	0.385	0.737 (0.670–0.797)	0.614	0.787	0.538	0.835
CKD stage								
IV	122/31	0.824 (0.739–0.909)	0.438	0.787 (0.704–0.856)	0.581	0.857	0.581	0.857
V	114/31	0.758 (0.662–0.855)	0.359	0.728 (0.637–0.807)	0.613	0.771	0.500	0.842

AUC, area under curve; CI, confidence interval; PPV, positive predictive value; NPV, negative predictive value; BMI, body mass index; mod-rig, moderate to rigor; CKD, chronic kidney disease.

### Subgroup performance analysis

The subgroup performance of the AITIS model, stratified by age, sex, BMI, physical exercise, occupation, and CKD stage, was evaluated in the study population (Table 2). Overall, the model maintained strong performance across the various subgroups investigated. Specifically, when considering the AUC as the reference metric for model performance, the model demonstrated relatively higher efficacy (defined as an AUC 0.025 higher than that of the total population) in patients who engage in less physical exercise (AUC = 0.843, 95% CI = 0.764–0.922) and in those patients with stage IV CKD (AUC = 0.824, 95% CI = 0.739–0.909).

### Feature importance and model explainability

**Figure 2:**
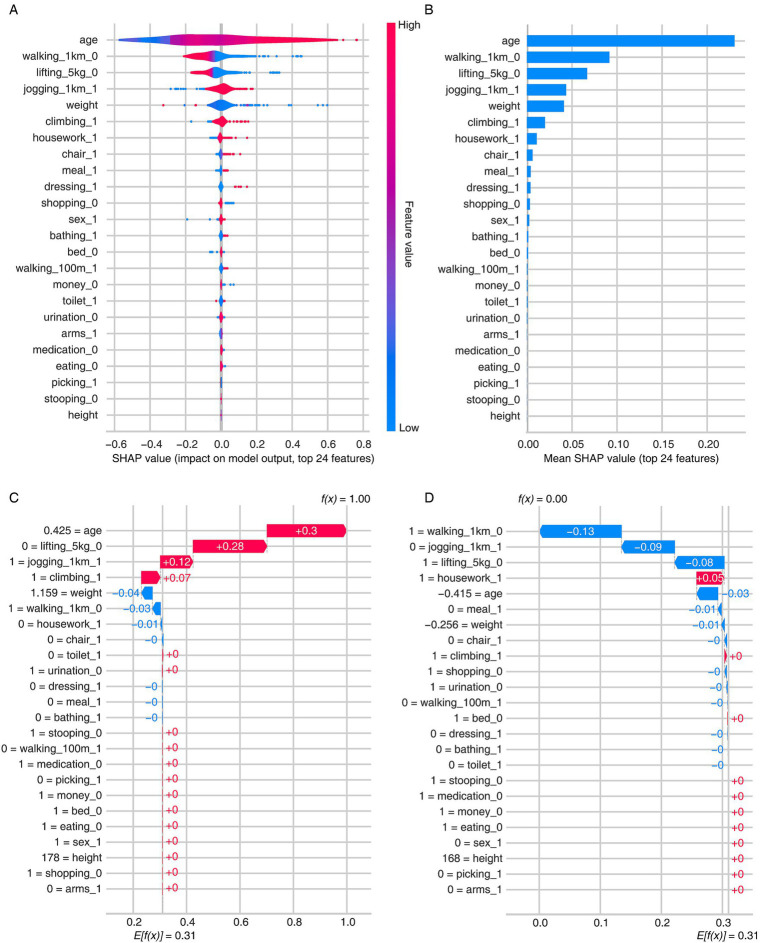
Model explainability using the SHapley Additive exPlanations (SHAP) method. **(A)** Group-level explainability using a violin plot. **(B)** Group-level explainability using a bar plot. **(C)** Individual-level explainability (high probability). **(D)** Individual-level explainability (low probability).

The feature importance and explainability of the AITIS model were evaluated using the SHAP method (Figure 2). The SHAP summary plot revealed that age, walking 1 km, and lifting 5 kg were the top three contributors to the high likelihood of sarcopenia (Figures 2A,B). We also examined individual-level risk predictions and identified their sources of risk as specified by the SHAP values. For the patient with the highest predicted SHAP value (e.g., 1), age (+0.30), lifting 5 kg (+0.28), and jogging 1 km (+0.12) were the leading sources of risk contributing to the high SHAP value (Figure 2C). Conversely, for the patient with the lowest SHAP value (e.g., 0), walking 1 km (−0.13), jogging 1 km (−0.09), and lifting 5 kg (−0.08) were the primary sources of risk leading to the low SHAP value (Figure 2D).

### Clinical usefulness

**Figure 3:**
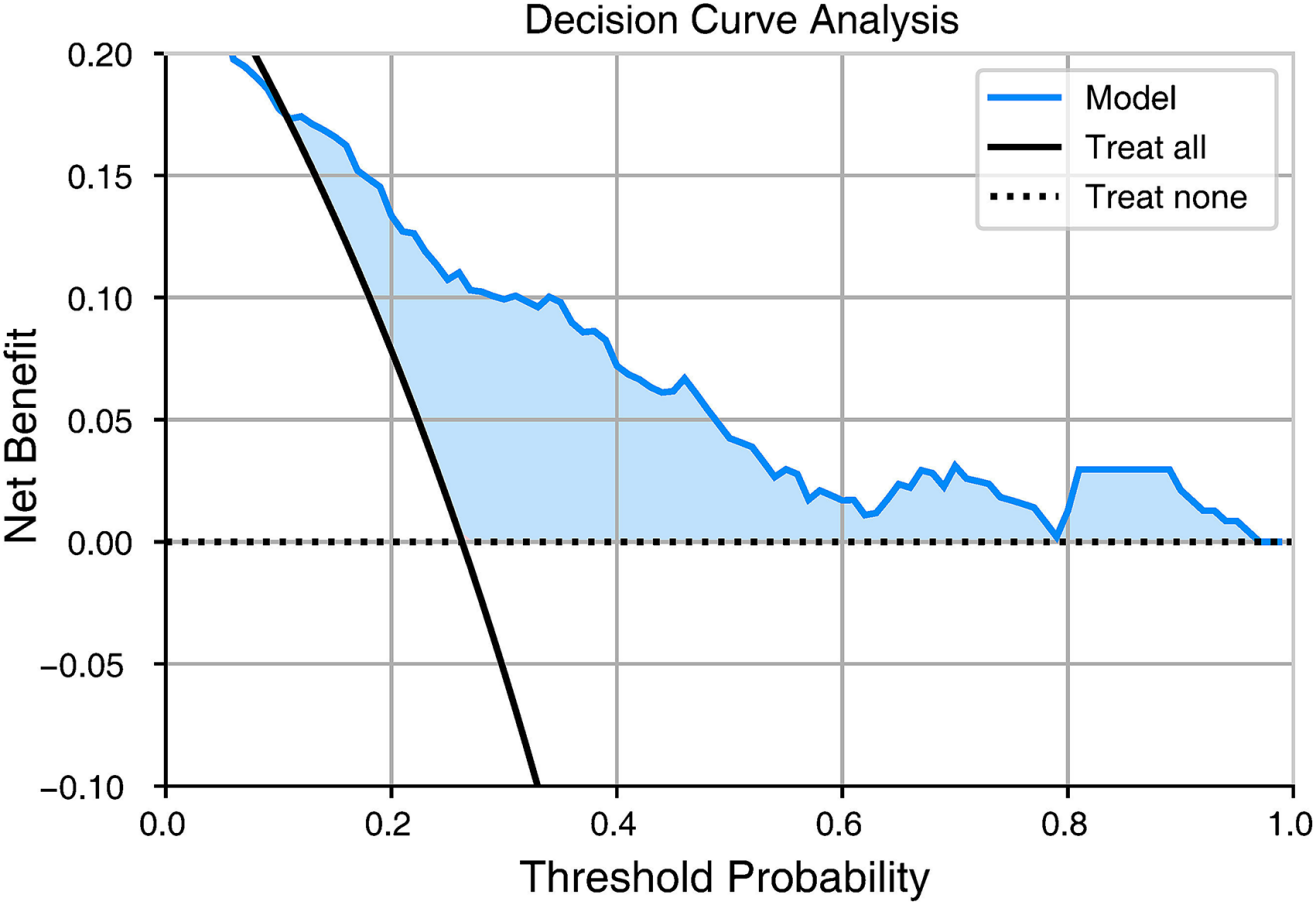
Decision curve analysis.

Decision curve analysis indicated that if the threshold probability for an individual was greater than 0.01, using the model to predict the probability of sarcopenia provided more benefits than either the treat-all-subjects scheme or the treat-none scheme (Figure 3).

## Discussion

This observational cross-sectional study included 236 non-dialysis patients with stage IV–V CKD at our center. It represents the first systematic clinical validation of AITIS, an AI model we previously developed. The key finding confirms that this model demonstrates feasibility (AUC = 0.792) in this population. AITIS utilizes simple input variables such as age, sex, weight, height, and FC-related questions that support self-reporting. Therefore, it is suitable for both clinicians and patients to monitor muscle status across different settings. This approach may save resources and time compared to conventional screening strategies that rely extensively on medical professionals and equipment. Our findings have the potential to assist clinicians in making informed decisions regarding the screening and surveillance of individuals at risk of sarcopenia, thereby guiding management strategies to enhance outcomes for CKD patients.

The prevalence of sarcopenia in the study population was 26.3%. This statistic is significantly higher compared to previous studies conducted in community-dwelling adults (9, 18). In the context of CKD, the prevalence of sarcopenia varies greatly across different studies. A prior study reported a prevalence of 7% for sarcopenia according to the Foundation for the National Institutes of Health (FNIH) and 5% according to the EWGSOP in patients with CKD (23). A meta-analysis indicated a prevalence of sarcopenia ranging from 3.89 to 69% in CKD patients (24, 25). Another meta-analysis reported a prevalence of 26.8% for sarcopenia in patients undergoing hemodialysis (HD) and 17.5% in those on peritoneal dialysis (PD) (13). Variations in the prevalence of sarcopenia in CKD may arise from differences in factors such as diagnostic criteria, geographic region or ethnic group, sex, and age. Despite unmodifiable factors such as age and sex, the diagnostic framework used to define sarcopenia may be the primary modifiable factor contributing to the variations in its prevalence. More importantly, the absence of a universal diagnostic framework hinders the implementation of standardized clinical management pathways and the aggregation of research findings. Recently, the Global Leadership Initiative in Sarcopenia (GLIS) was proposed to harmonize competing definitions and establish a diagnostic gold standard for sarcopenia (8). Future studies adopting the GLIS framework may help reduce the heterogeneity in the prevalence of sarcopenia across studies.

In the subgroup analysis, we observed that the model had the best performance in patients who engage in less regular physical exercise (Table 2). One possible explanation is that the AITIS model was trained on a cohort that included middle-aged and older adults. Individuals in this age group may have less structured exercise compared to younger adults. Consequently, the model was trained with a greater number of instances from this subgroup, resulting in better fitting within it. Another possible explanation is that patients who engage in less regular physical exercise may experience more difficulties with activities of daily living (such as jogging 1 km or lifting 5 kg). In this scenario, the model can better establish a mapping between physical measures and outcome labels during the inference process, leading to more accurate predictions. However, we observed no univariate association between physical exercise and sarcopenia (*p* = 0.400), implying that other mechanisms may underlie this result. Subsequent studies should investigate this further within CKD populations, particularly those with more extensive physical exercise data, to replicate our findings.

Sarcopenia in patients with CKD is largely treatable. A UK study found that ensuring adequate dietary protein intake is effective in preventing lean body mass loss in CKD patients receiving PD. (26) Another randomized, double-blind, placebo-controlled trial conducted with Chinese patients undergoing dialysis also found that sarcopenia can be partially reversed through integrative medicine approaches (27). A recent network meta-analysis provides compelling evidence that both exercise and nutritional interventions may improve muscle mass, strength, and/or function in HD patients (28). These findings underscore the value of the present study, which has the potential to support the early identification of sarcopenia, facilitating timely interventions in patients with CKD. Future studies may explore whether referring patients identified as having a high probability of sarcopenia, as predicted by the AITIS model, to intervention programs could more effectively reverse or alleviate the progression of sarcopenia in at-risk patients who have not yet met the diagnostic criteria for sarcopenia.

The SHAP analysis has revealed some interesting results worth commenting on. Among the top five FC measures that contributed to sarcopenia prediction, three primarily involve the lower limb musculoskeletal system (walking 1 km, jogging 1 km, and climbing stairs). This aligns with our recent study showing that CST optimally reflects the diversity and magnitude of daily activities individuals perform (29). CST was proposed as a simple measure of lower-limb muscle strength (30), which has been extensively adopted in clinical practice and diagnostic frameworks for sarcopenia (9, 10). Lower-limb strength is inherently prioritized in functional assessments due to its direct association with an individual’s mobility. Additionally, objective measures of physical performance (e.g., usual gait speed, stair-climb power test, 6-min walk test, CST, timed up-and-go test, and short physical performance battery) predominantly evaluate the lower limb musculoskeletal system (9, 31–34). This evidence suggests that, compared to an upper-limb-focused strategy, the identification and intervention of sarcopenia in CKD may need to concentrate on the lower limb musculoskeletal system due to its superior role in improving functional outcomes related to sarcopenia status. The GLIS framework also introduced a new criterion for diagnosing sarcopenia: muscle-specific strength, defined as muscle strength standardized to muscle size (8). Our research findings, along with existing evidence, suggest that the diagnosis of sarcopenia is evolving toward a more refined approach.

The strength of this study lies in several key aspects. The foremost aspect is that it used BIA to measure ASMI and establish the diagnosis of sarcopenia (outcome label), which is recommended by international guidelines (9, 10) and is expected to be more accurate than the AITIS study that used an anthropometric equation to estimate ASMI (18). Nevertheless, future studies should employ more accurate ASM assessment methods, such as DEXA, to validate our results. Second, this study performed further subgroup analyses to evaluate potential effect modifications, providing greater insights into the capabilities of the AITIS model and inspiring future research. For instance, future studies could investigate whether physical exercise (and its types) could modify the association between functional disability and sarcopenia.

However, several potential limitations and issues regarding generalizability must be noted. First, compared to other geographic or ethnic groups, Asians may exhibit differences in muscle and functional measures (9, 10). Future studies need to replicate our findings in other populations, such as Caucasian individuals. Second, sarcopenia diagnoses were made using the AWGS 2019 framework, and other diagnostic frameworks were not evaluated. Third, while our sample size meets the requirements for preliminary validation, it may not fully represent the broader CKD population, including patients at earlier stages of CKD. Therefore, the generalizability to other patient groups requires further assessment. However, a previous review found that the global prevalence of sarcopenia in CKD did not differ among stages (11), which suggests that our findings may be generalizable to patients with earlier stages. Fourth, in our preliminary experiments, we used an alternative modeling strategy by splitting the 236 study samples into training (*n* = 165) and validation (*n* = 71) sets to redevelop the model. However, this led to model overfitting and poor generalizability in the validation set (data not shown). Additionally, the original AITIS model was developed using a cohort of 8,162 individuals (18), which already included a greater number of patients with CKD compared to the present study (20). For the reasons above, we chose to validate the original AITIS model instead of developing a new one. Future studies with larger sample sizes and *de novo* model development in CKD may further improve the model’s performance. Fifth, despite the model’s overall good performance, the heterogeneity in performance revealed by subgroup analysis should be regarded as an important caution for precise clinical application. Future studies with larger sample sizes, as well as multicenter and prospective designs, should address these issues.

In conclusion, we clinically validated an artificial intelligence model, the AITIS, in non-dialysis patients with stage IV–V CKD. AITIS utilizes a unique test-free, self-assessable design, demonstrating clinical feasibility in predicting sarcopenia in this population. These findings may advance the scientific basis for adopting AITIS in the CKD context and inform targeted sarcopenia prevention and intervention strategies to optimize patient outcomes.

## Data Availability

The datasets presented in this article are not readily available due to the protection of patient confidentiality. Requests to access the datasets should be directed to LY, liangyuyin1988@tmmu.edu.cn.

## Footnotes

1
https://xinqiaohospital-don-lyy-sarco.streamlit.app

2
https://github.com/kevinlyy/sarco

## References

[1] Romagnani P Remuzzi G Glassock R Levin A Jager KJ Tonelli M . Chronic kidney disease. Nat Rev Dis Primers. (2017) 3:17088. doi: 10.1038/nrdp.2017.88, 29168475

[2] Kovesdy CP. Epidemiology of chronic kidney disease: an update 2022. Kidney Int Suppl. (2022) 12:7–11. doi: 10.1016/j.kisu.2021.11.003, 35529086 PMC9073222

[3] Wang L Xu X Zhang M Hu C Zhang X Li C . Prevalence of chronic kidney disease in China: results from the Sixth China Chronic Disease and Risk Factor Surveillance. JAMA Intern Med. (2023) 183:298–310. doi: 10.1001/jamainternmed.2022.6817, 36804760 PMC9941971

[4] Zhang L Wang F Wang L Wang W Liu B Liu J . Prevalence of chronic kidney disease in China: a cross-sectional survey. Lancet. (2012) 379:815–22. doi: 10.1016/S0140-6736(12)60033-6 22386035

[5] Zoccali C Mark PB Sarafidis P Agarwal R Adamczak M de Bueno Oliveira R . Diagnosis of cardiovascular disease in patients with chronic kidney disease. Nat Rev Nephrol. (2023) 19:733–46. doi: 10.1038/s41581-023-00747-4 37612381

[6] Kidney Disease: Improving Global Outcomes (KDIGO) CKD Work Group. KDIGO 2024 clinical practice guideline for the evaluation and management of chronic kidney disease. Kidney Int. (2024) 105:S117–314. doi: 10.1016/j.kint.2023.10.018 38490803

[7] Okamura M Konishi M Butler J Kalantar-Zadeh K von Haehling S Anker SD. Kidney function in cachexia and sarcopenia: facts and numbers. J Cachexia Sarcopenia Muscle. (2023) 14:1589–95. doi: 10.1002/jcsm.13260, 37222019 PMC10401526

[8] Kirk B Cawthon PM Arai H Avila-Funes JA Barazzoni R Bhasin S . The conceptual definition of sarcopenia: Delphi consensus from the Global Leadership Initiative in Sarcopenia (GLIS). Age Ageing. (2024) 53:afae052. doi: 10.1093/ageing/afae052, 38520141 PMC10960072

[9] Chen LK Woo J Assantachai P Auyeung TW Chou MY Iijima K . Asian Working Group for Sarcopenia: 2019 consensus update on sarcopenia diagnosis and treatment. J Am Med Dir Assoc. (2020) 21:300–307.e2. doi: 10.1016/j.jamda.2019.12.012, 32033882

[10] Cruz-Jentoft AJ Bahat G Bauer J Boirie Y Bruyere O Cederholm T . Sarcopenia: revised European consensus on definition and diagnosis. Age Ageing. (2019) 48:601. doi: 10.1093/ageing/afz046, 31081853 PMC6593317

[11] Duarte MP Almeida LS Neri SGR Oliveira JS Wilkinson TJ Ribeiro HS . Prevalence of sarcopenia in patients with chronic kidney disease: a global systematic review and meta-analysis. J Cachexia Sarcopenia Muscle. (2024) 15:501–12. doi: 10.1002/jcsm.13425, 38263952 PMC10995263

[12] Hung R Wong B Goldet G Davenport A. Differences in prevalence of muscle wasting in patients receiving peritoneal dialysis per dual-energy X-ray absorptiometry due to variation in guideline definitions of sarcopenia. Nutr Clin Pract. (2017) 32:539–44. doi: 10.1177/0884533617696331, 28760111

[13] Wathanavasin W Banjongjit A Avihingsanon Y Praditpornsilpa K Tungsanga K Eiam-Ong S . Prevalence of sarcopenia and its impact on cardiovascular events and mortality among dialysis patients: a systematic review and meta-analysis. Nutrients. (2022) 14:4077. doi: 10.3390/nu14194077, 36235729 PMC9572026

[14] Wang L Zhu B Xue C Zhou F Luo Q. Sarcopenia is associated with mortality among newly initiated peritoneal dialysis patients with end-stage kidney disease: a longitudinal follow-up study. BMC Nephrol. (2025) 27:17. doi: 10.1186/s12882-025-04664-5, 41340104 PMC12781612

[15] Sabatino A Guerra A Baiocchi de Carvalho KM Cuppari L Carrero JJ Stenvinkel P . Sarcopenia and its individual traits independently predict mortality in patients on dialysis: a systematic review and meta-analysis. J Cachexia Sarcopenia Muscle. (2025) 16:e70089. doi: 10.1002/jcsm.70089, 41069070 PMC12510902

[16] Garrido-Ardila EM Castro Lemus MA Ramirez-Duran MDV Jimenez-Palomares M Martin Hidalgo-Barquero MV Gonzalez-Sanchez B . Effects of exercise on sarcopenia and frailty in haemodialysis patients: a systematic review. Medicina. (2025) 61:2204. doi: 10.3390/medicina61122204 41470206 PMC12734946

[17] Hu X Wu B Yang Y Zhang L Xue C. Sarcopenia in peritoneal dialysis: prevalence, pathophysiology, and management strategies. Kidney Med. (2025) 7:100989. doi: 10.1016/j.xkme.2025.100989, 40247955 PMC12005912

[18] Yin L Zhao J. An artificial intelligence approach for test-free identification of sarcopenia. J Cachexia Sarcopenia Muscle. (2024) 15:2765–80. doi: 10.1002/jcsm.13627, 39513334 PMC11634523

[19] Zhao Y Hu Y Smith JP Strauss J Yang G. Cohort profile: the China Health and Retirement Longitudinal Study (CHARLS). Int J Epidemiol. (2014) 43:61–8. doi: 10.1093/ije/dys203, 23243115 PMC3937970

[20] Cao Y Tang M Zhao J Yin L. Association of combined left and right handgrip strength with new-onset chronic kidney disease in middle-aged and older adults: a nationwide multicenter cohort study. BMC Public Health. (2025) 25:988. doi: 10.1186/s12889-025-22149-w, 40082839 PMC11905662

[21] Yin L Li F Xiao T Zhang J Li Y Luo J . Functional performance decline outperforms sarcopenia and its components in predicting new-onset chronic kidney disease: a nationwide multicenter study. Kidney Med. (2025) 7:101005. doi: 10.1016/j.xkme.2025.101005, 40510605 PMC12152322

[22] Chen C Lu FC Department of Disease Control Ministry of Health, PR China. The guidelines for prevention and control of overweight and obesity in Chinese adults. Biomed Environ Sci. (2004) 17:1–36. 15807475

[23] Bellafronte NT Goveia TR Chiarello PG. Sarcopenia in chronic kidney disease: prevalence by different definitions and relationship with adiposity. Appl Physiol Nutr Metab. (2022) 47:915–25. doi: 10.1139/apnm-2021-0521, 35658617

[24] Huang YF Liu SP Muo CH Lai CY Chang CT. Male sex and ageing are independent risk factors for sarcopenia stage in patients with chronic kidney disease not yet on dialysis. J Cachexia Sarcopenia Muscle. (2024) 15:2684–92. doi: 10.1002/jcsm.13612, 39351998 PMC11634482

[25] Jin K Li X Ma Y Yang D Tan X Sun Q . Risk factors associated with sarcopenia in patients with chronic kidney disease: a systematic review and meta-analysis. J Cachexia Sarcopenia Muscle. (2026) 17:e70166. doi: 10.1002/jcsm.70166, 41457350 PMC12745342

[26] Davenport A. Does lower dietary protein intake result in lower muscle mass in patients with end-stage kidney failure treated by peritoneal dialysis? A retrospective study. Nutr Clin Pract. (2025) 41:530–9. doi: 10.1002/ncp.11346, 40605430

[27] Li X Wang R Lu K He L Li L Yong R . Efficacy and safety of Shen-Qi paste, a traditional Chinese medicine, in dialysis patients with sarcopenia: a randomized, double-blind, placebo-controlled trial. Phytomedicine. (2025) 147:157190. doi: 10.1016/j.phymed.2025.157190, 40886476

[28] Zhang L Wang F Tashiro S Liu PJ. Nutritional and exercise interventions for muscle status in hemodialysis patients: a systematic review and network meta-analysis. Clin Nutr. (2025) 53:35–48. doi: 10.1016/j.clnu.2025.08.005, 40845422

[29] Yin L Zhao J. Objective measures of physical functioning, disabilities in daily life and trends during ageing: a repeated cross-sectional study. J Cachexia Sarcopenia Muscle. (2025) 16:e70133. doi: 10.1002/jcsm.70133, 41277015 PMC12641453

[30] Csuka M McCarty DJ. Simple method for measurement of lower extremity muscle strength. Am J Med. (1985) 78:77–81. doi: 10.1016/0002-9343(85)90465-6, 3966492

[31] Ramsey KA Meskers CGM Maier AB. Every step counts: synthesising reviews associating objectively measured physical activity and sedentary behaviour with clinical outcomes in community-dwelling older adults. Lancet Healthy Longev. (2021) 2:e764–72. doi: 10.1016/S2666-7568(21)00203-8, 36098033

[32] Studenski S Perera S Patel K Rosano C Faulkner K Inzitari M . Gait speed and survival in older adults. JAMA. (2011) 305:50–8. doi: 10.1001/jama.2010.1923, 21205966 PMC3080184

[33] Zanker J Sim M Anderson K Balogun S Brennan-Olsen SL Dent E . Consensus guidelines for sarcopenia prevention, diagnosis and management in Australia and New Zealand. J Cachexia Sarcopenia Muscle. (2023) 14:142–56. doi: 10.1002/jcsm.13115, 36349684 PMC9891980

[34] Amaral Gomes ES Ramsey KA Rojer AGM Reijnierse EM Maier AB. The association of objectively measured physical activity and sedentary behavior with (instrumental) activities of daily living in community-dwelling older adults: a systematic review. Clin Interv Aging. (2021) 16:1877–915. doi: 10.2147/CIA.S326686, 34737555 PMC8560073

