# Supplementary material for: Clinical utility of the AITIS model for test-free identification of sarcopenia in patients with stage IV–V non-dialysis-dependent chronic kidney disease
Source: Front Nutr. 2026 Apr 28;13:1793383. doi: 10.3389/fnut.2026.1793383 (PMC13160844; doi:10.3389/fnut.2026.1793383)
Supplement: Supplementary file 1 [file Data_Sheet_1.PDF]

Supplementary files

Number	Description
1	**Table S1.** Functional capacity items included for analysis in the present study
2	**Figure S1.** A flowchart of the patient inclusion

Table S1. Functional capacity indices included for analysis in the present study

Category	Index	Detailed description
ADL	Dressing	Do you have some difficulty with dressing?
	Bathing	Do you have some difficulty with bathing?
	Eating	Do you have some difficulty with eating?
	Bed	Do you have some difficulty with getting in and out of bed?
	Toilet	Do you have some difficulty with using the toilet?
	Urination	Do you have some difficulty with controlling urination and defecation?
IADL	Money	Do you have some difficulty with managing money?
	Medication	Do you have some difficulty with taking medications?
	Shopping	Do you have some difficulty with shopping for groceries?
	Meal	Do you have some difficulty with preparing meals?
	Housework	Do you have some difficulty with cleaning house?
Other	Jogging 1km	Do you have some difficulty with running or jogging 1km?
	Walking 1km	Do you have some difficulty with walking 1km?
	Walking 100m	Do you have some difficulty with walking 100m?
	Chair	Do you have some difficulty with getting up from a chair after sitting for long periods?
	Climbing	Do you have some difficulty with climbing several flights of stairs without resting?
	Stooping	Do you have some difficulty with stooping, kneeling, or crouching?
	Lifting 5kg	Do you have some difficulty with lifting or carrying weights over 5kg?
	Picking	Do you have some difficulty with picking up a coin from the table?
	Arm	Do you have some difficulty with reaching arms above shoulder level?

Abbreviations: ADL, activities of daily living; IADL, instrumental activities of daily living.

Figure S1

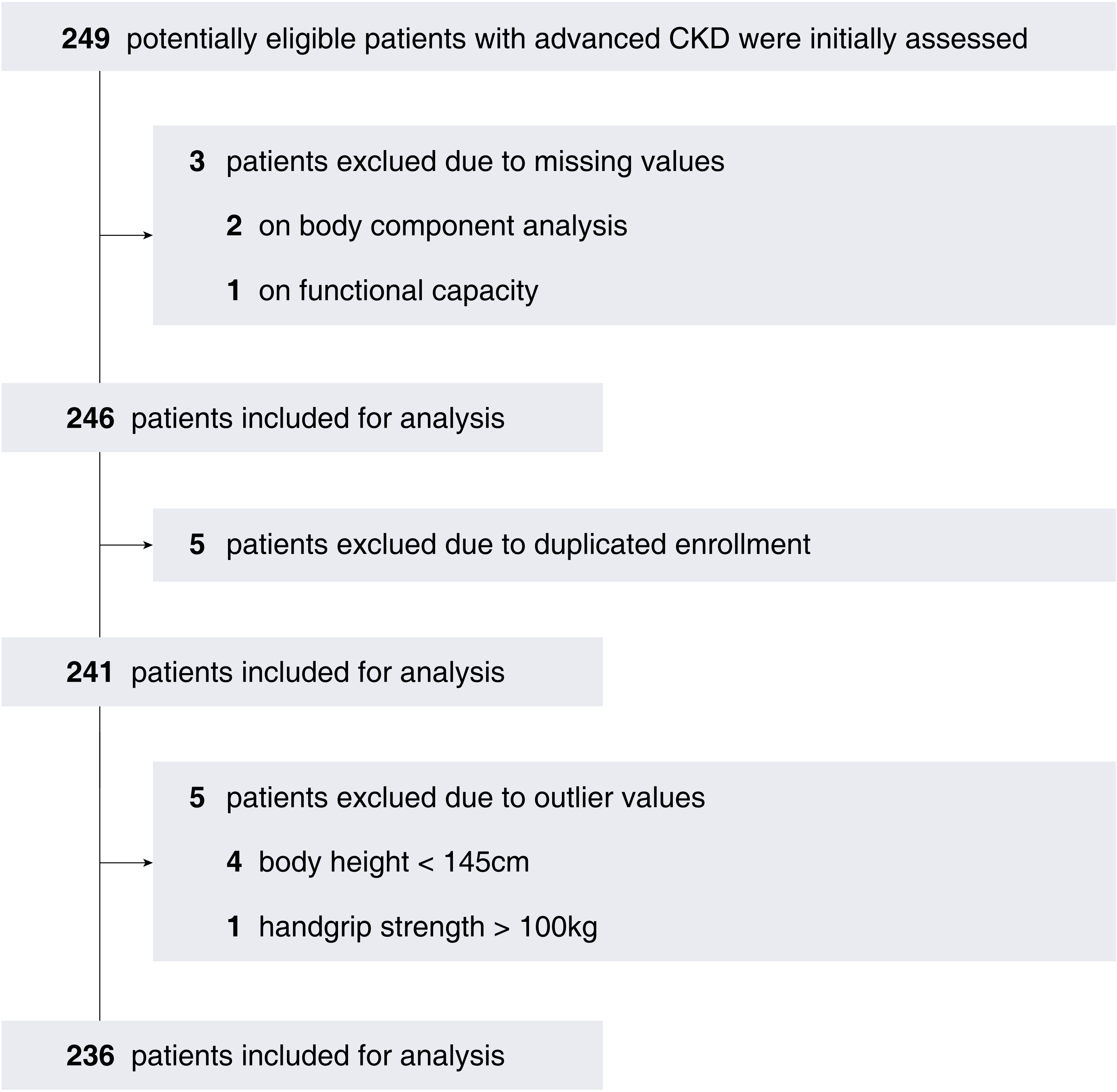

249 potentially eligible patients with advanced CKD were initially assessed
3 patients exclued due to missing values
2 on body component analysis
1 on functional capacity
246 patients included for analysis
5 patients exclued due to duplicated enrollment
241 patients included for analysis
5 patients exclued due to outlier values
4 body height < 145cm
1 handgrip strength > 100kg
236 patients included for analysis
